# Post-gastric Sleeve Surgery Chronic Symptoms From a Sample of Patients in Saudi Community

**DOI:** 10.7759/cureus.42000

**Published:** 2023-07-17

**Authors:** Waleed M Alhuzaim, Raghad M Alajlan, Rahaf A Alshehri, Razan M Alanazi, Leen K Alsarhan, Hala K Alamri

**Affiliations:** 1 Gastroenterology, Imam Mohammad Ibn Saud Islamic University, Riyadh, SAU; 2 Medicine, Imam Mohammad Ibn Saud Islamic University, Riyadh, SAU

**Keywords:** abdominal symptoms, psychological symptoms, musculoskeletal/neurological symptoms, gastroesophageal reflux disease (gerd), symptoms, gastric sleeve surgery

## Abstract

Background: A common bariatric procedure known as gastric sleeve surgery can cause significant weight loss and co-morbid condition alleviation. However, patients could experience persistent problems such as gastrointestinal, musculoskeletal/neural, and psychiatric disorders after surgery. This study aims to identify the most prevalent chronic symptoms following sleeve gastrectomy among a sample of Saudi patients and the impact these symptoms have on patients' lives.

Methodology: Patients who underwent gastric sleeve surgery at the Ensan Clinic, a facility specializing in gastroenterology, were the subjects of this retrospective cohort analysis. The study population consisted of patients who underwent gastric sleeve surgery, showed up for follow-up after the procedure, and met the inclusion and exclusion criteria. The data collection sheet is divided into seven sections. Sociodemographic information was required in the first section, gastric sleeve surgery information in the second, vital signs in the third, lab results in the fourth, past medical history in the fifth, current treatments in the sixth, and postoperative complications and chronic symptoms in the seventh and final sections.

Results: In 117 patients, the study evaluated the effects of gastric sleeve surgery. Participants had an average age of 40.21 years, and 61.5% were female. Regarding persistent symptoms after surgery, a sizable percentage of patients mentioned digestive issues such as GERD (44.4%), dyspepsia (60.7%), vomiting (23.1%), nausea (39.3%), and abdominal distention (45.3%). A total of 34.2% of patients reported experiencing anxiety, compared to 11.1% who said they had depression or 2.6% who said they had social issues. A few patients reported experiencing neurological or musculoskeletal issues, including exhaustion (7.7%), faintness (5.1%), back or joint discomfort (7.7%), and shortness of breath (8.5%).

Conclusion: After undergoing gastric sleeve surgery, a sizable proportion of patients complained of various chronic symptoms and nutritional inadequacies, primarily gastrointestinal problems and musculoskeletal/neurological issues. The study's findings show a connection between these symptoms and surgery.

## Introduction

Obesity is characterized by an anomalous or excessive accumulation of body fat that may have adverse health effects [1]. According to the World Health Organization's (WHO) classification of adult obesity, a body mass index (BMI) between 25 and 29.9 kg/m^2^ is considered overweight, whereas a BMI greater than 30 kg/m^2^ is considered obese [2]. The worldwide increase in obesity prevalence is one of the most significant contemporary threats to public health. The estimated national weighted prevalence of obesity (BMI 30 kg/m^2^) in Saudi Arabia 2021 was 24.7% [3]. Surgical weight loss has been shown to be a reliable and effective treatment for morbidly obese individuals [4,5]. In the past, laparoscopic sleeve gastrectomy (LSG) was considered a possible first-stage therapy for obese patients; however, it is now commonly performed as a stand-alone bariatric procedure for high-risk and profoundly obese patients [4].

Surgical treatment for morbid obesity has significantly evolved since the invention of laparoscopy. As a treatment option for morbid obesity, various surgeries with a wide spectrum of modifications are currently recommended [6]. LSG has become popular as the primary procedure for morbidly obese patients to lose weight [7]. The standard principle of restriction underlies sleeve gastrectomy, which also entails the removal of orexigenic cells that release the hormone ghrelin from the fundus of the stomach [8].

Ren and his colleagues performed the first laparoscopic sleeve gastrectomy in 1999. LSG was considered a first-stage procedure for high-risk patients [9]. Although there are several published studies on the efficacy of LSG for long-term weight loss, they pale in comparison to the persuasive data regarding short- and midterm outcomes [10]. Consequently, this study aims to identify the most prevalent chronic symptoms following sleeve gastrectomy among a sample of Saudi patients, as well as the impact these symptoms have on patients' lives.

## Materials and methods

Study design and population

In Riyadh, Saudi Arabia, a retrospective cohort analysis of patients who underwent gastric sleeve surgery at a clinic specializing in gastroenterology (Ensan Clinic) was conducted. The study population consisted of patients who underwent gastric sleeve surgery, presented for follow-up after the procedure, and met the inclusion and exclusion criteria.

Inclusion criteria included having undergone gastric sleeve surgery, being at least 18 years old, being of either gender and presenting with complete data for follow-up. Those without follow-up and those younger than 18 were excluded from the study. There were no other exclusion criteria regarding patients' previous functional pathologies.

Data collection

The relevant data were collected using a data collection form. The co-authors manually filled out each case's form. The data collection document contains seven sections. The first section required sociodemographic information, the second section information about gastric sleeve surgery, the third section vital signs, the fourth section laboratory results, the fifth section past medical history, the sixth section current treatments, and the final section postoperative complications and chronic symptoms.

Statistical analysis

Excel was utilized for data entry, data cleaning, and coding. The data were then encoded and transferred to SPSS 23 for analysis. Frequency and percentages were used in displays of categorical variables. Mean, and standard deviation was utilized for presenting continuous variables.

Ethical approval

Throughout the investigation, all data were handled with the utmost discretion, and privacy was maintained. Ethical approval was requested from the ethical council of Imam Mohammad Ibn Saud Islamic University. Due to the study's retrospective nature and the use of anonymized patient data, the ethical committee recommended waiving the informed consent requirement.

## Results

A total of 117 participants who underwent gastric sleeve surgery were included in the study. The average age of the patients was 40.21 (SD = 11.5) years, and the preponderance was female (61.5%). The average weight before surgery was 113.3 kg (SD=23.7), and the average weight after surgery was 87.1 kg (SD=64.6), resulting in a mean weight loss of 31.7 kg (SD=19.0). The average years since surgery was 4.0 (standard deviation = 2.6).

Following surgery, 18.8 percent of patients had a BMI within the normal range, 52.1% were overweight, and 29.0% were obese. Compared to 86.3% of patients with normal blood pressure, 5.1% with high blood pressure, and 7.9% with hypertension, only 0.9% had low blood pressure. Most patients (94.9%) had a normal heart rate, while 2.6% had a low heart rate, and 2.6% had a high heart rate.

A total of 10.3% of patients reported smoking, while 89.7% reported not smoking. Table 1 shows that 32.5% of patients reported having undergone additional abdominal surgeries, while 67.5% reported never having endured such procedures.

**Table 1:** Baseline patients’ characteristics BMI: Body Mass Index, SD: Standard deviation

		Count	%
Age	Mean (SD)	40.2	11.5
Weight (Before surgery) (Kg)	Mean (SD)	113.3	23.7
Weight (After surgery) (Kg)	Mean (SD)	87.1	64.6
Weight loss (Kg)	Mean (SD)	31.7	19.6
Duration since surgery ( years)	Mean (SD)	4.0	2.6
BMI (Post surgery)	Underweight	0	0.0%
	Normal weight	22	18.8%
	Overweight	61	52.1%
	Obese	34	29.1%
Blood pressure (mmHg)	Low	1	0.9%
	Normal	101	86.3%
	High	6	5.1%
	Hypertension	9	7.7%
Heart rate (beats per minute)	Normal	111	94.9%
	Low	3	2.6%
	High	3	2.6%
Smoking	Yes	12	10.3%
	No	105	89.7%
Other Abdominal surgery	Yes	38	32.5%
	No	79	67.5%

The proportion of patients with normal laboratory examinations following gastric sleeve surgery is presented in Table 2. Vitamin B12, vitamin D, calcium, iron, ferritin, hemoglobin, blood sugar, and urea level were measured in the laboratory.

**Table 2:** Proportion of patients with normal laboratory findings after the surgery

	Count	%
Vitamin B 12 (μg)	41	35.0%
Vitamin D (μg)	35	29.9%
Calcium level (mmol/L)	38	32.5%
Iron level (μmol/L)	34	29.1%
Ferritin level (ng/mL)	33	28.2%
Hemoglobin level (g/dL)	56	47.9%
Blood sugar level (mmol/L)	36	30.8%
Urea level (mmol/L)	52	44.4%

The past medical history and medications used by patients who underwent gastric sleeve surgery are presented in Table 3. In the past medical history, obstructive sleep apnea syndrome (OSAS), diabetes, hypertension, and dyslipidemia were present. Among the medications used after surgery were antihypertensive drugs, proton pump inhibitors (PPIs), insulin/weight-reduction injections, diet control alone, oral agents for type 2 diabetes (T2D), statins for dyslipidemia, and continuous positive airway pressure (CPAP) for OSAS. Based on their medical history, the majority of individuals did not have diabetes mellitus (88.9%), hypertension (93.2%), dyslipidemia (99.2%), or OSAS (100%). Very few patients had a history of these diseases. The majority of patients did not use antihypertensive medications (94.9%), insulin/weight-reduction injections (97.5%), statins for dyslipidemia (98.1%), or CPAP for OSAS (99.1%). After surgery, however, a substantial proportion of patients utilized PPIs (55.6%), diet control alone (20.5%), or oral T2D medications (6.9%).

**Table 3:** Past medical history and post-surgery used medications CPAP: continuous positive airway pressure, OSAS: Obstructive Sleep Apnea Syndrome, PPIs: Proton Pump inhibitors, T2D: Type 2 diabetes

		Count	%
Past medical history	Diabetes mellitus (Yes)	13	11.1%
	Hypertension (Yes)	8	6.8%
	Dyslipidemia (Yes)	1	0.9%
	OSAS (Yes)	0	0.0%
Post-surgery used medications	Antihypertensive drugs	6	5.1%
	PPIs	65	55.6%
	Insulin/weight reducing injection	3	2.6%
	Diet control only	24	20.5%
	Oral agents for T2D	8	6.8%
	Statins for dyslipidemia	1	0.9%
	CPAP for OSAS	1	0.9%

Individuals who have undergone gastric sleeve surgery have been observed to exhibit the symptoms listed in Table 4. The symptoms were categorized as musculoskeletal/neurological, gastrointestinal, psychological, and others. Regarding musculoskeletal/neurological complaints, a negligible proportion of patients reported weakness (3.4%), lethargy (7.7%), vertigo (5.1%), and back/joint pain (7.7%). No patients reported paresthesia, dysesthesia, hypoesthesia, or degenerative joint disease symptoms. Concerning gastrointestinal symptoms, a significant proportion of patients reported having GERD (44.4%), dyspepsia (60.7%), vomiting (23.1%), nausea (39.3%), eating difficulties (6.8%), abdominal pain (41%), dysphagia (9.4%), diarrhea (14.5%), constipation (22.2%), peptic ulcer (2.6%), or abdominal distension (45.4%). Few patients (6.8%) reported experiencing acid reflux. Concerning psychiatric symptoms, a considerable proportion of patients (34,2%) reported suffering from anxiety, while a smaller proportion (11.1%) reported experiencing depression or social problems (2.6%). Regarding other symptoms, a substantial proportion of patients (12.8%) had their gallbladder removed, whereas a smaller proportion (3.4%) had gallstones. Significantly more patients encountered hernias (19.7 percent) than hair loss (4.3%), dehydration (0%), shortness of breath (8.5%), or difficulty sleeping (3.4%).

**Table 4:** Proportion of patients with post-gastric sleeve surgery chronic symptoms GERD: Gastroesophageal reflux Diseases, SOB: Shortness of breath

	Count	%	
Musculoskeletal/Neural symptoms			
Weakness	4		3.4%
Lethargy	9		7.7%
Dizziness	6		5.1%
Back/joint pain	9		7.7%
Gastrointestinal symptoms:			
GERD	52		44.4%
Dyspepsia	71		60.7%
Heartburn	8		6.8%
Vomiting	27		23.1%
Nausea	46		39.3%
Eating problems	8		6.8%
Abdominal pain	48		41.0%
Dysphagia	11		9.4%
Diarrhea	17		14.5%
Constipation	26		22.2%
Peptic ulcer	3		2.6%
Abdominal distension	53		45.3%
Psychiatric Symptoms			
Depression	13		11.1%
Anxiety	40		34.2%
Social problems	3		2.6%
Other symptoms			
Gall bladder			
Normal	98	83.8%	
Removed	15	12.8%	
Stone	4	3.4%	
Hernias	23		19.7%
Sleeping problem	4		3.4%
SOB	10		8.5%
Hair loss	5		4.3%

## Discussion

Obesity is a serious global health issue, and bariatric surgery has become a common treatment option for individuals who are morbidly obese [11]. Gastric sleeve surgery, one of the most common bariatric procedures, entails removing a portion of the stomach to reduce its size and restrict food intake [12]. Although gastric sleeve surgery has demonstrated positive results in weight loss and management of comorbidities, postoperative complications, and chronic pain may occur. This study aimed to investigate the laboratory results and chronic symptoms of individuals who had undergone gastric sleeve surgery.

According to laboratory examinations, most postoperative patients had normal blood sugar, hyperuricemia, and hemoglobin levels. Nonetheless, a substantial proportion of patients undertaking gastric sleeve surgery had low levels of vitamin B12, vitamin D, calcium, iron, and ferritin, highlighting the necessity of monitoring and managing these laboratory markers. These results are consistent with previous reports [13-16] indicating that patients who have undergone gastric sleeve surgery frequently experience dietary deficiencies. Possible causes of deteriorating laboratory results include insufficient dietary intake, malabsorption, and inadequate supplementation [17]. After gastric sleeve surgery, the stomach is shrunk, reducing the quantity of food that can be consumed [18]. Patients must ingest nutrient-dense foods in order to meet their nutritional needs. Malabsorption can also develop due to a smaller stomach size and altered intestinal structure [15]. As patients may not be meeting their dietary requirements, inadequate vitamin and mineral supplementation may also be a factor. A deficiency in vitamin B12 can result in anemia, fatigue, and nerve damage, while a deficiency in vitamin D can result in bone loss and an increased risk of fractures [19]. Low calcium levels can cause bone loss, muscle spasms, and nerve injury, while low iron levels can cause anemia and fatigue. Low ferritin levels can also result in anemia and fatigue [17]. Vitamin and mineral supplements may be necessary for patients who undergo gastric sleeve surgery to prevent nutritional deficiencies.

The results indicated that patients may develop gastrointestinal, musculoskeletal/neurological, mental, and other chronic complications following surgery. Consistent with prior research [20-24] indicating a high prevalence of gastrointestinal symptoms following gastric sleeve surgery, a substantial proportion of patients reported experiencing GERD, dyspepsia, regurgitation, nausea, abdominal discomfort, and abdominal distension. GERD is characterized by the reflux of stomach contents into the esophagus [25]. Heartburn and regurgitation are symptoms of GERD. Indigestion, or dyspepsia, is characterized by upper abdominal distress or pain, bloating, and early satiety [26]. Vomiting and nausea are common postoperative symptoms that may result from changes in gastrointestinal motility and hormone communication. Abdominal pain and distension may be linked to changes in gastrointestinal structure and microbiome [26]. Changes in gut bacteria decreased gastric acid production, and altered gut hormone signaling may contribute to gastrointestinal symptoms following surgery [27]. The stomach is dramatically reduced in size after gastric sleeve surgery, resulting in changes to the gut microbiome and gut hormone signaling [27]. These alterations may affect the digestion and absorption of food, resulting in gastrointestinal issues [27]. Reduced stomach acid production may also contribute to GERD and dyspepsia, as stomach acid aids digestion and eliminates harmful gut bacteria [17]. Preexisting gastrointestinal diseases like irritable bowel syndrome (IBS) and inflammatory bowel disease (IBD) may also contribute to post-surgery gastrointestinal complications [28]. Patients must address any preexisting gastrointestinal issues with their healthcare providers before undergoing gastric sleeve surgery. Adjustments to the diet, such as consuming smaller meals and avoiding foods that may provoke symptoms, as well as medications to alleviate symptoms, such as acid reducers and anti-nausea drugs, may be used to treat post-surgery gastrointestinal problems [29]. In exceptional cases, revisional surgery may be required to treat postoperative complications.

In addition to gastrointestinal issues, many patients reported musculoskeletal/neurological complaints, such as back/joint pain and shortness of breath, which may be related to changes in body composition and physical activity induced by weight loss [30]. Changes in body composition and physical activity brought on by weight loss may contribute to musculoskeletal distress after surgery [31]. Extreme weight loss may cause joint alignment and body mass distribution changes, resulting in increased joint and back tension [32]. As a result of changes in body composition and physical activity caused by weight loss, shortness of breath may also occur. After a significant weight loss, a person's lung capacity and function may alter, resulting in physical exertion-related shortness of breath [33]. Psychiatric disorders may also contribute to musculoskeletal and neurological complications after surgery [34], in addition to anxiety and depression. After surgery, patients may experience increased stress and emotional anguish, resulting in increased muscular tension and discomfort. In addition, anxiety and depression may affect the quality and quantity of sleep, resulting in increased fatigue and lethargy [35, 36]. Musculoskeletal and neurological disorders may be treated with physical therapy, exercise, and painkillers. Additionally, resolving any underlying psychological disorders, such as anxiety and depression, may aid in managing these symptoms [37].

Many patients also reported psychiatric symptoms, such as anxiety and depression, which may be related to the emotional and psychological difficulties associated with bariatric surgery [37]. Bariatric surgery is a life-altering procedure that can significantly impact a patient's mental health. Due to the surgical procedure and the difficulties of adjusting to a new lifestyle, such as dietary and social adjustments, patients may experience anxiety and depression [37]. In addition, patients may experience anxiety and hopelessness due to the fear of regaining weight and the potential for postoperative complications [38]. In addition to alterations in hormone signaling and neurotransmitter function, psychiatric symptoms following surgery may also be associated with alterations in hormone signaling. Changes in gastrointestinal hormone signaling after gastric sleeve surgery may affect mood and appetite regulation [39,40]. Changes in intestinal flora following surgery may also affect the production of mood-regulating neurotransmitters, such as serotonin [41]. Psychotherapy, behavioral therapy, and medication may all be utilized to treat mental symptoms. Additionally, treating any underlying psychological issues, such as anxiety and depression, may be beneficial for managing these symptoms [40].

Before undergoing a specific therapeutic intervention or medical treatment, individuals may experience physical or emotional suffering. Regarding post-gastric sleeve surgery chronic symptoms, pre-therapeutic sufferings can be defined as "the problems and obstacles individuals encounter prior to deciding to undergo gastric sleeve surgery for weight-related concerns" [42,43]. Before deciding to perform gastric sleeve surgery, individuals frequently endure various pre-therapeutic afflictions related to obesity and associated health issues. These can include physical distress such as difficulty performing daily activities, sleep apnea, and an increased risk of various medical conditions such as hypertension, diabetes, and heart disease. In addition, emotional and psychological distress can result from low self-esteem, body image issues, societal stigmatization, and a negative effect on the overall quality of life [42-43].

Individuals who have undergone gastric sleeve surgery may experience chronic symptoms which are long-lasting or persistent. Although gastric sleeve surgery can be an effective weight loss method, some patients may experience chronic symptoms after the procedure. Depending on the individual, these symptoms may include nausea, vomiting, abdominal discomfort, acid reflux, and alterations in bowel habits [44]. Pre-therapeutic suffering can affect how individuals perceive and manage post-gastric sleeve chronic symptoms. Individuals may initially view surgery as a potential remedy for their preexisting mental and physical afflictions. They may anticipate that the surgery will mitigate these issues and enhance their overall health. However, if a patient develops post-surgical chronic symptoms, it can be difficult for them to navigate and manage these new obstacles. This individual's mental and emotional state may be affected by the presence of these chronic symptoms, which may cause further distress or frustration [42-43].

Individuals seeking gastric sleeve surgery should thoroughly comprehend the procedure's potential pros and cons. They should have reasonable expectations regarding the outcomes and be aware of the possibility of postoperative complications and persistent symptoms. To address these concerns, individuals should engage in in-depth conversations with their healthcare providers and endure exhaustive pre-operative evaluations. This enables medical professionals to evaluate the patient's overall health, identify any pre-existing conditions that may impact surgery, and provide adequate guidance and support throughout the process.

Limitations of the study

This study is limited by its retrospective methodology, which may introduce recollection bias and impede its ability to establish causality. In addition, the research was conducted in a singular clinic, which may limit its applicability to other populations. The sample size is comparatively small, and the duration of follow-up is limited to four years, which may not represent long-term outcomes. In addition, the lack of a control group hinders the ability to compare the outcomes of patients who did and did not undergo gastric sleeve surgery. Physical activity, dietary behaviors, and mental health status may be crucial factors influencing weight loss and other outcomes after gastric sleeve surgery, but the study did not collect data on these variables.

Recommendation

Based on the findings of this study, several recommendations can be made to enhance the outcomes for individuals who undergo gastric sleeve surgery. To prevent deficiencies in vitamin B12, vitamin D, calcium, iron, and ferritin, patients should first receive nutritional monitoring and supplementation regularly. Medical personnel must provide adequate guidance and support to ensure that patients meet their nutritional requirements. Second, patients with a history of gastrointestinal issues should undergo a thorough preoperative evaluation to determine their suitability for surgery and receive recommendations for dietary modifications and medications to treat postoperative gastrointestinal symptoms. Thirdly, patients with prolonged postoperative symptoms should receive the appropriate treatment, including physical therapy, analgesics, counseling, and medications to treat underlying psychological issues. Fourthly, patients should receive comprehensive education and support before and during gastric sleeve surgery in order to assist them in adjusting to the resultant lifestyle changes. Additional research is necessary to examine the long-term effects of gastric sleeve surgery on nutritional deficiencies and chronic complaints and the impact of preconception assessment and postoperative management on patient outcomes. These recommendations highlight the importance of a comprehensive preoperative evaluation, postoperative management of chronic complaints, and patient education and support in ensuring optimal outcomes for patients undergoing gastric sleeve surgery.

## Conclusions

A significant proportion of patients who underwent gastric sleeve surgery in Riyadh, Saudi Arabia, reported suffering from various chronic symptoms and nutritional deficiencies, primarily gastrointestinal and musculoskeletal/neurological symptoms. The study's findings indicate a connection between these symptoms and surgery. Therefore, postoperative care, such as routine monitoring of chronic symptoms and nutritional supplementation, is crucial to the long-term success of the surgery. Additional research is required to investigate the underlying causes of the complications and chronic symptoms and develop effective management strategies.
